# Headaches in the elderly, in an out-patient population over 60 years of age

**DOI:** 10.1186/1129-2377-1-S14-P229

**Published:** 2013-02-21

**Authors:** E Venturelli, R Rao, S Gipponi, P Liberini, A Padovani

**Affiliations:** 1 Neurological Clinic Headache Center Spedali Civili University of Brescia, Italy

## 

Headache in community-living adults age 65 and older is the 10th most common reported symptom in women and the 14th most common in men. Although the prevalence of headache declines with age, approximately 10% of women and 5% of men at age 70 experience severe recurrent or constant headaches. Much less is known about the evolving clinical profile of migraine over the life span. The present study aimed to investigate every type of headaches in elderly people and was carried out on a group of patient over 60 years of age, selected from 771 consecutive patients to the Headache Centre in the period January 2011-December 2011.

## Methods

This study was conducted in a university-based outpatient headache clinic. The study population consisted of 771 consecutive headache patients treated by the authors in one year. Variables studied included gender, headache duration in years, aura, headache characteristics, associated symptoms, presence of allodynia, headache frequency, headache days, and disability. Amedical history of these patients was also recorded. The headache diagnosis were made according to ICHD-2 criteria. Patients were stratified by age into 3 groups: group 1, 16 to 39, group II, 40 to 59, and group III, 60 years and older.

## Results

A total of 605 patients were female and 166 were male, mean age was 36.9 + 13.6 years (range 16 to 84), average headache duration 18.4 years, and headache days/month 7.9. The average age of older headache suffers was 66.5 years. There were 48 female patients (7.9%) and 6 male patients (3.6%) in the older age group. There were no differences between the groups in gender and other variables assessed. The 60+ age group tended to have more chronic migraine and to use more acute medication. Discussion In our population chronic migraine and medication overuse don’t decline over time. We found that, compared with younger patients, older headache patients had not a “lesser migraine” as reported in previous studies. Studies of community-based headache population are warranted to define the influence of age on the full spectrum of migraine.
